# Association between tricuspid annular systolic velocity and poor short-term prognosis in patients with acute decompensated heart failure

**DOI:** 10.1080/07853890.2022.2134585

**Published:** 2022-10-19

**Authors:** Kai Zhao, Jiang Zhou, Jing Tao Guo, Cai Hong Wu, Sen Lin Li, Qi Zhang, Xiang Tian, Wei Chao Shan, Zhen Jiang Ding, Lan Suo Yuan, Qun Zheng, Xiao Li Gao, Nan Guo, Hong Sen Tian, Qing Min Wei, Xi Tian Hu, Ying Kai Cui, Xue Geng, Qian Wang, Wei Cui

**Affiliations:** a Department of Cardiology, The Second Hospital of Hebei Medical University, Shijiazhuang, China; b Department of Cardiology, Chengde Central Hospital, Chengde, China; c Department of Cardiology, First Hospital of Zhangjiakou, Zhangjiakou, China; d Department of Cardiology, First Central Hospital of Baoding, Baoding, China; e Department of Cardiology, Affiliated Hospital of Chengde Medical University, Chengde, China; f Department of Cardiology, Hengshui People’s Hospital, Hengshui, China; g Department of Cardiology, Huabei Petroleum Administration Bureau General Hospital, Cangzhou, China; h Department of Cardiology, Cangzhou Central Hospital, Cangzhou, China; i Department of Cardiology, Handan Central Hospital, Handan, China; j Department of Cardiology, Xingtai People’s Hospital, Xingtai, China; k Department of Cardiology, Shijiazhuang People’s Hospital, Shijiazhuang, China; l Department of Cardiology, The 252nd Hospital of People’s Liberation Army, Baoding, China

**Keywords:** Tricuspid annular, systolic velocity, prognosis, acute decompensated heart failure

## Abstract

**Background:**

There is scant data on the association of the Pulsed wave-Doppler tissue imaging (PW-DTI)-derived tricuspid lateral annular peak systolic velocity (S’) and poor short-term prognosis of patients with acute decompensated heart failure (ADHF).

**Patients and methods:**

A total number of 732 participants from the Heb-ADHF registry in China were divided into three groups according to the corresponding status of tricuspid S′. Demographic characteristics, comorbidities, physical examinations, lab tests, and medications were compared among the different groups. Different logistic regression models were utilized to gauge the relationship between S′ and the risk of a composite of short-term all-cause mortality or 30-day heart failure (HF)-related rehospitalization.

**Results:**

The number of composite outcome events identified in the study population was 85, with the short-term all-cause death coupled with 30-day HF readmission events reaching 23 and 62, respectively. As per the multivariable adjusted analysis, S′ was inversely related to the risk of a composite outcome [<10 cm/s odds ratios (OR) 2.90, 95% confidence interval (CI):1.33–6.31; 10–11 cm/s OR 2.18, 95% CI: 1.10–4.33; *p* for trend = 0.006] in comparison with S′ at >11 cm/s. When S′ was analysed as a continuous variable, per 1 cm/s increase, the OR (95% CI) for a composite outcome was [0.87 (0.77–0.99), *p* = 0.028]. Area under curve (AUC) of S′ for predicting outcome of ADHF was 0.631 (95%CI: 0.573**–**0.690, *p* < 0.01). Significant inverse association was also observed in left ventricular ejection fraction (LVEF) ≥40% subgroup.

**Conclusions:**

Inspite of the potential confounders, a more impaired tricuspid annular peak systolic velocity is associated with a poorer short-term prognosis of patients with ADHF.

## Key message

- This is the first comprehensive evaluation of tricuspid annular systolic velocity among patients with ADHF.
- Tricuspid annular systolic velocity could be a predictor of poor short-term prognosis in ADHF.
- Tricuspid annular systolic velocity should be considered in patients with ADHF at admission.

## Introduction

Acute decompensated heart failure (ADHF) is characterized by a relatively swift change in the signs or symptoms of heart failure (HF), thus leading to unscheduled therapy or hospitalization [1,2]. Burgeoning evidence indicates that the natural history of ADHF is featured by a marked increase in recurrent post-discharge events as opposed to high in-hospital mortality especially within 3 months, which renders this condition as one of leading causes for hospitalization in elderly patients both in developed and developing countries [3–8]. However, a growing number of studies have demonstrated that in spite of the unremitting efforts to reduce rehospitalizations for ADHF, there appears to be only a slight impact on the corresponding readmissions [9,10]. Therefore, clinicians or researchers should particularly fixate on factors associated with the undesirable recent prognosis of ADHF in order to identify the high-risk patients and subsequently provide more tailored as well as comprehensive strategies at preventing further cardiovascular events.

Several investigations have implicated that the right ventricular (RV) dysfunction is linked to poor outcomes across a broad spectrum of disorders, such as myocardial infarction, chronic HF, and pulmonary hypertension (PH) [11–17]. However, there seems to be a dearth of study on the association between the RV dysfunction and unfavourable short-term prognosis of ADHF. Although there are several available approaches to evaluate the RV function, such as cardiac magnetic resonance imaging (CMR), radionuclide ventriculography, and right heart catheterization, it seems basically impossible to apply them in clinical practice due to the high cost, radiation exposure, and invasiveness. Notably, Pulsed wave-Doppler tissue imaging (PW-DTI)-derived tricuspid lateral annular peak systolic velocity (S′) occurs as one of the multiple parameters applied for the assessment of RV systolic function due to its easy operability, reproducibility, and noninvasiveness. To date, S′ has been validated to reveal a specific correlation with other measurements of global RV systolic function, with its abnormality threshold (<9.5 cm/sec) equivalent to that of RV systolic dysfunction according to recommendations from American society of echocardiography [18–20]. However, there is a scare study on the prognosis of patients with low level of normal range of S′ (i.e. 10 or 11 cm/sec).

In summary, we performed a retrospective analysis to assess the relevance of S′ in the poor short-term prognosis of ADHF.

## Patients and methods

### Study population

The Heb-ADHF (Hebei-acute decompensated heart failure) study (ChiCTR-POC-17014020) is a prospective, multicenter, and observational study, which enrolled patients discharged from hospitals with a main diagnosis of HF in accordance with Chinese HF guideline [1]. The study was approved by the human research ethics committee of the second hospital of Hebei medical university (2015110) and is in conformity to the ethical standards of the institutional and/or national research committee and 1964 Helsinki declaration as well as its later amendments or comparable ethical standards.

### Ethics, consent and permissions

Verbal informed consents were obtained from all patients included in the study.

### Consent to publish

We have obtained consent to publish from the participant to report individual patient data.

ADHF was defined as a de novo acute heart failure (AHF) or decompensation of chronic heart failure (CHF). Inclusion criteria include: (1) age ≥18 y; (2) unplanned admission; (3) with typical symptoms or signs of ADHF; and (4) brain natriuretic peptide (BNP) levels >100 pg/mL or N-terminal pro-brain natriuretic peptide (NT-proBNP) levels >300 pg/mL. Exclusion criteria include: (1) hospital stay <24 h; (2) heart transplantation; (3) on renal replacement therapy; (4) massive stroke; (5) concomitant terminal disease; or (6) patients lost to follow-up.

We collected substantial data on the participants’ baseline characteristics including age, sex, body mass index (BMI), details of first admission, New York Heart Association (NYHA) or Killip functional class, (If both grading methods are applicable to a patient with acute myocardial infarction, choose the more severe one), comorbidities including coronary artery disease (CAD), acute myocardial infarction (AMI), hypertension, and valvular heart disease (VHD), and records of physical examinations, laboratory tests, and baseline medications. Each variable is based on the European Society of Cardiology (ESC)recommendations on HF initial evaluation [21].

**Figure 1.**
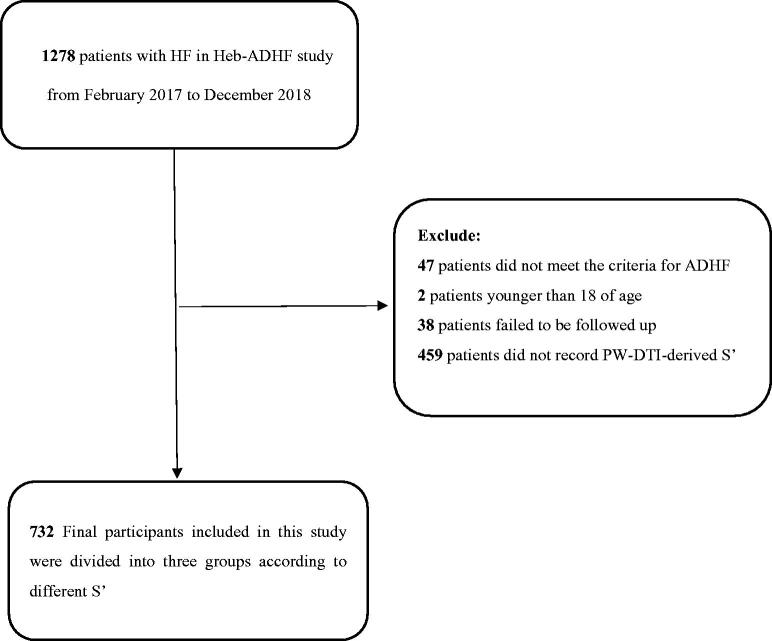
Total Heb-ADHF population, exclusions and the final study populations. *Abbreviations*: Heb-ADHF: Hebei-acute decompensated heart failure; ADHF: acute decompensated heart failure; PW-DTI: pulsed wave-Doppler tissue imaging.

To explore the correlation between the S′ wave velocity and adverse recent prognosis of ADHF, participants were chosen from the Heb-ADHF registry after the corresponding exclusions. A flowchart describing the details of patient selection is shown in Figure 1.

### Echocardiographic parameters and pre-defined S′ intervals

All patients underwent transthoracic echocardiograph exams using the iE33 (Philips Medical Systems-Andover, MA, USA) apparatus with 5 and 8 MHz transducers during the in-hospital period. The apical, parasternal and subcostal views were used to acquire the M-mode, 2-dimensional and color-, continuous- and PW-DTI data in light of the American Society of Echocardiography. We gathered considerable echocardiographic parameters (initial assessment) corresponding to the left and right sides of the heart. The former includes left ventricular ejection fraction (LVEF, assessed using Simpson’s method or Teichholz formula), early diastolic mitral septal annular velocity (e′ septal), ratio of mitral peak velocity of early filling (E) to early diastolic mitral septal annular velocity (e′ septal) (E/e′ septal ratio), left atrial (LA) diameter, left ventricular end-diastolic diameter (LV EDD), and left ventricular end-diastolic volume (LV EDV). On the other hand, the latter consists of right atrial (RA) diameter, right ventricular (RV) diameter along with pulmonary arterial systolic pressure (PASP) that is gauged using a Doppler echocardiograph [right atrial pressure(RAP)+4*tricuspid regurgitant velocity (TRV)^2^], PH(PASP >40 mmHg), and pulmonary artery (PA) diameter. S′ was used to quantify RV systolic function and was obtained from a RV- focussed apical view as the Doppler beam parallelly aligned with RV free wall longitudinal excursion. Baseline characteristics were compared among the three groups of patients according to S′ tertiles: <10 cm/s (group 1, low), 10–11 cm/s (group 2, intermediate), and >11 cm/s (group 3, high). The group 3 (>11 cm/s) was reckoned as a reference for statistical analysis. S′<10 cm/s was defined as RV systolic dysfunction on the strength of recommendation from American society of echocardiography [20].

### Follow-up and outcomes

Each recruited participant was followed up for 30 days after discharge through a telephonic interview by a trained staff. Data on events of death or readmission were collected at each follow-up. The primary outcome was a composite of all-cause mortality within 30 days after discharge (including in-patient) or 30-day HF rehospitalization.

### Statistical analysis

Baseline characteristics were summarized in each interval of S′. Moreover, the distribution of each quantitative variable was evaluated using the Shapiro-Wilk test. Normally distributed and homogenous data were described as mean ± standard deviation (SD), and the discrepancies among the three groups were gauged using the one-way analysis of variance (ANOVA). On the contrary, variables were represented based on the medium and interquartile range (IQR). Additionally, the Kruskal–Wallis test was performed for the comparison of the measured data of skewed distribution following the appropriate *post-hoc* test (pairwise comparisons). Categorical variables were reported as numbers and percentages (%) and were analyzed using the Pearson’s Chi square test or Fisher’s exact test. Pairwise comparisons were done using Bonferroni’s corrections.

Multivariable logistic regression models were implemented to eliminate impacts of confounders, while sensitivity analyses were utilized to ensure data robustness. Baseline variables that were clinically correlated or that presented a univariable relevance to the risk of a composite outcome with a *p*-value <0.1 were entered into the multivariable logistic regression models (additional details are presented in Online Resource Table 1). Variables for inclusion were cautiously spotted, in consideration of the number of events available, to ensure the parsimony of final models (events per variable ≥ 5).

**Table 1.** Baseline characteristics of three groups of participants stratified by different S′.

Characteristics	Level	Group 1 (S′: <10cm/s) (*n* = 181)	Group 2 (S′: 10–11cm/s) (*n* = 290)	Group 3 (S′: >11cm/s) (*n* = 261)	*p* Values
Age(years)	Median (IQR)	61 (48–68)	68 (59–75)*	67 (58–75)*	<0.001
Male	*n* (%)	131 (72.4)	160 (55.2)*	166 (63.6)	0.001
BMI (kg/m^2^)	Median (IQR)	24.4 (21.6–26.6)	24.2 (22.0–26.8)	24.8 (22.8–26.8)	0.301
missing	*n* (%)	49 (27.1)	73 (25.2)	63 (24.1)	
First admission	*n* (%)	60 (33.1)	112 (38.6)	145 (55.6)*,§	<0.001
Smoking	*n* (%)	33 (18.2)	38 (13.1)	48 (18.4)	0.173
NYHA class	*n* (%)				<0.001
II		16 (8.8)	48 (16.6)*	52 (19.9)*	
III		87 (48.1)	163 (56.2)	130 (49.8)	
IV		72 (39.8)	62 (21.4)*	54 (20.7)*	
Killip class					
II		4 (2.2)	14 (4.8)	22 (8.4)*	
III		2 (1.1)	3 (1.0)	3 (1.1)	
IV		0	0	0	
Comorbidity	*n* (%)				
CAD		79 (43.6)	156 (53.8)*	157 (60.2)*	0.003
AMI		5 (2.8)	17 (5.9)	36 (13.8)*,§	<0.001
Hypertension		76 (42.0)	158 (54.5)*	170 (65.1)*,§	<0.001
VHD		34 (18.8)	56 (19.3)	43 (16.5)	0.669
DCM		70 (38.7)	40 (13.8)*	25 (9.6)*	<0.001
DM		38 (21.0)	72 (24.8)	64 (24.5)	0.598
Stroke/TIA		22 (12.2)	41 (14.1)	52 (19.9)	0.056
A fib/A flutter		56 (30.9)	122 (42.1)	64 (24.5)§	<0.001
CKD		11 (6.1)	25 (8.6)	15 (5.7)	0.360
Physical examinations	Median (IQR)				
Heart rate (bpm)		88 (75–100)	83 (70–100)	80 (68–91)*	0.002
SBP (mmHg)		117 (105–133)	125 (112–140)*	131 (116–150)*,§	<0.001
DBP (mmHg)		78 (70–89)	79 (71–89)	79 (69–89)	0.969
Laboratory tests	Median (IQR)				
BNP (pg/ml)		1450 (658–2500)	722 (333–1405)*	566 (340–1175)*	<0.001
Missing	*n* (%)	32 (17.7)	53 (18.3)	36 (13.8)	
NT-proBNP (pg/ml)		6858 (3345–13,919)	5445 (2501–7826)	4593 (2534–7290)	0.059
Missing	*n* (%)	136 (75.1)	228 (78.6)	217 (82.8)	
eGFR (mL/min/1.73 m2)		75 (60–90)	78 (57–92)	80 (64–98)	0.054
FBG (mmol/L)		5.1 (4.4–6.0)	5.2 (4.6–6.3)	5.1 (4.5–6.4)	0.447
Missing	*n* (%)	12 (6.6)	19 (6.2)	14 (5.0)	
Serum albumin (g/L)		39 (36–41)	39 (36–42)	39 (35–41)	0.212
Serum potassium (mmol/L)		4.1 (3.6–4.4)	4.0 (3.7–4.4)	4.0 (3.7–4.3)	0.652
Serum sodium (mmol/L)		141 (138–143)	141 (138–143)	142 (139–143)	0.799
Baseline medications	*n* (%)				
Loop diuretics		178 (99.4)	278 (95.9)*	248 (95.0)*	0.026
Thiazide diuretics		8 (4.6)	20 (6.9)	14 (5.4)	0.504
ACEI/ARBs		94 (51.9)	139 (47.9)	151 (57.9)	0.066
β-Blockers		148 (81.8)	211 (72.8)	166 (63.6)*	<0.001
MRA (spirolactone)		163 (90.1)	250 (86.2)	217 (83.1)	0.133
Statin		85 (47.0)	161 (55.5)	153 (58.6)*	0.036

BMI: body mass index; NYHA: New York Heart Association; CAD: coronary artery disease; AMI: acute myocardial infarction; VHD: valvular heart disease; DCM: dilated cardiomyopathy; DM: diabetes mellitus; TIA: transient ischaemic attack; A fib/A flutter: atrial fibrillation/atrial flutter; CKD: chronic kidney disease; SBP: systolic blood pressure; DBP: diastolic blood pressure; LVEF: left ventricular ejection fraction; BNP: brain natriuretic peptide; NT-pro BNP: N-terminal pro brain natriuretic peptide; eGFR: estimated glomerular filtration rate; eGFR is calculated using the MDRD eGFR equation as follows: eGFR(mL/min/1.73m^2^)=186* [serum creatinine (ummol/L)/88.4)]-1.154*(age)-0.203*(0.742 if female); FBG: fasting blood glucose: ACEI/ARBs: angiotensin-converting enzyme inhibitors/angiotensin receptor blockers; MRA: mineralocorticoid receptor antagonist. Percentages may not total 100 because of rounding.

*p < 0.017 vs. group1.

^§^*p* < 0.017 vs. group2.

Odds ratios (OR) and 95% confidence intervals (CI) were calculated to assess the relationship between S’ and risk of a composite outcome. Notably, each quantitative variable demonstrated a liner correlation with the logit transformation on outcome using the Box-Tidwell method. In addition, multi-collinearity did not exist among all the confounding factors according to the upshots of variance inflation factors (VIF, all < 10; correlation coefficients between each two variables were less than 0.7). the multivariable model was first adjusted for age and sex (model 1) and then further adjusted for first admission, NYHA class IV, hypertension, dilated cardiomyopathy (DCM), A fib/A flutter, serum sodium, serum albumin, SBP, and β-blockers (model 2). The model was re-adjusted for echocardiographic parameters containing LVEF, LV EDD, RA diameter, and RV diameter (model 3, fully adjusted model). We also conducted tests to assess the linear trend by entering the median value of each tertile of S′ as a continuous variable in corresponding models.

To maximize the statistical power and minimize bias, we implemented multiple imputation (MI), which was based on 5 replications and the Markov-chain Monte Carlo method in the SPSS MI procedure, to account for the missing data on the PASP/PH and E/e′ [22]. This led to all the missing data that was fully imputed in the study population. We repeated all the analyses with the complete and multiple imputed data for comparison. The Online Resource Table 2 presents the additional details of the statistical analyses.

**Table 2.** Echocardiographic parameters of three groups of patients.

Variable	Level	Group 1 (S′: <10cm/s) (*n* = 181)	Group 2 (S′: 10-11cm/s) (*n* = 290)	Group 3 (S′: >11cm/s) (*n* = 261)	*p* Values
Left heart	Median (IQR)				
LV EDD (mm)		63 (54–70)	53 (47–62)*	53 (47–60)*	<0.001
LV EDV (ml)		189 (141–240)	127 (94–179)*	124 (101–171)*	<0.001
LA diameter (mm)		46 (42–50)	42 (39–48)*	40 (37–45)* §	<0.001
LVEF (%)		31 (26–41)	45 (34–61)*	54 (40–61)* §	<0.001
LVE*F* < 40%	*n* (%)	134 (74.0)	108 (37.2)*	64 (24.5) * §	<0.001
e′ septal (cm/s)		4 (3–4)	4 (3–5)*	5 (4–6) * §	<0.001
Missing	*n* (%)	11 (6.1)	24 (8.3)	13 (5.0)	
E/e′ septal		24 (18–31)	19 (14–27)*	17 (14–21) * §	<0.001
Missing	*n* (%)	11 (6.1)	24 (8.3)	13 (5.0)	
AO diameter (mm)		31 (28–34)	31 (28–34)	31 (29–34)*	0.017
Right heart	Median (IQR)				
RA diameter (mm)		42 (36–48)	37 (33–43)*	36 (33–40)*	<0.001
RV diameter (mm)		28 (22–45)	22 (20–29)*	21 (20–24)* §	<0.001
S′ (cm/s)		8 (7–9)	10 (10–11)*	13 (12–14)* §	<0.001
PASP (mmHg)		49 (34–67)	40 (30–54)*	36 (28–50)*	<0.001
missing	*n* (%)	24 (13.3)	49 (16.9)	64 (24.5)	
PAS*p* >40mmHg (%)		103 (65.6)	117 (48.5)*	85 (43.1)*	<0.001
PA diameter (mm)		28 (26–32)	27 (25–30)*	27 (25–30)*	0.006

EDD: end-diastolic diameter; EDV: end-diastolic volume; LA: left atrial; LVE: left ventricular ejection fraction; AO: aortic; RA: right atrial; RV: right ventricular; PASP: pulmonary arterial systolic pressure; PA: pulmonary artery.

*p < 0.017 vs. group 1.

^§^*p* < 0.017 vs. group 2.

We performed sensitivity analyses to ensure the data robustness as follows: we applied the LA size, not imputed or multiple imputed PH/PASP, and E/e′ data into model 3 for further adjustment, respectively, as each of them was clinically relevant or presented a univariable relevance to the risk of outcome with a *p*-value <0.1. Multivariable logistic regression models were similarly performed in the sensitivity analyses.

The ability to predict a composite outcome was evaluated using the area under the curve (AUC) in the receiver operating characteristic (ROC) curve. Using the Z test, we also compared the predictive accuracy of S’ with other echocardiographic parameters.

All analyses were conducted using SPSS version 25 (SPSS Inc., Chicago, IL, USA) and the AUROC curves were conducted using Medcalc software (version 20.0.3). A 2-sided *p* value <0.05 was considered to be statistically significant.

## Results

A total of 732 participants (62.4% men; median age 66 years) were categorized in S′ tertiles as low (group 1, S′:<10 cm/s), intermediate (group 2, S′: 10–11 cm/s), and high (group 3, S′: >11 cm/s), where each group consisted of 181, 290, and 261 patients, respectively.

### Characteristics and echocardiographic parameters of the study participants

In summary, patients in group 1 tended to be younger, had a lower prevalence of CAD and hypertension, decreased SBP, and a less frequent use of stains than the other groups. In contrast, the patients in groups 2 and 3 saw a reduced proportion of DCM and lower concentrations of BNP along with a more significant decline of β-blockers usage than group 1. As for echocardiographic parameters, participants with S′ below 10 cm/s had larger atrial and ventricular dimensions, lower LVEF, higher pulmonary arterial systolic pressure, and decreased values of e’ septal apart from raised upshots of E/e′ septal than the rest of the groups. Detailed baseline characteristics of all participants are summarized in Tables 1 and 2.

### *Relationship between S*′ *and poor short -term prognosis of ADHF*

**Figure 2.**
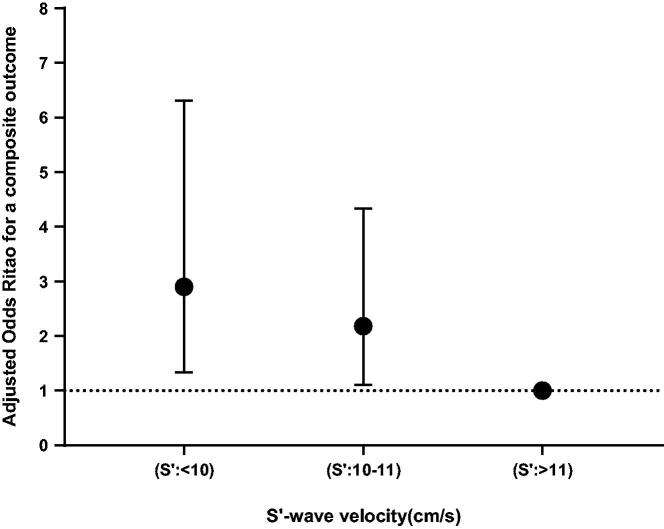
Final adjusted odds ratios with the 95% confidence interval for a primary outcome in all patients with ADHF.

We identified 85 composite outcome events within the 30 days of follow-up on every single discharged participant of the study population. The in-patient all-cause mortality of our present study was 2.3%, which is similar to the upshot observed in a previous study [23]. Meanwhile, the all-cause readmission rate at 30 days reached 10.1%, which was slightly lower than that of a previously reported formal trial [24]. Detailed information is presented in Table 3. The univariable and multivariable analyses (Table 4) demonstrated the OR (95% CI) for the risk of a composite outcome based on the alterations in S′ depending on whether each one was a categorical variable (tertiles) or a continuous variable (median value of each tertile). In contrast to the participants in group 3 (S′>11 cm/s), there was a significant increased risk of outcome with participants in group 1 (S′<10 cm/s; OR, 2.90; 95% CI, 1.33–6.31) alongside patients in group 2 (S′: 10–11 cm/s; OR, 2.18; 95% CI, 1.10–4.33; *p* for trend = 0.006) after the full adjustment for potential confounders that include age, sex, first admission, NYHA class IV, hypertension, DCM, A fib/A flutter, serum sodium, serum albumin, SBP, β-blockers, LVEF, LV EDD, RA diameter, and RV diameter. When RV-S′ was analysed as a continuous variable, the association between S’ and the risk of poor short-term prognosis in patients with ADHF maintained significantly in model 1, model 2 as well as model 3; and per 1 cm/s increase, the OR (95% CI) for a composite outcome was [0.87 (0.77–0.99), *p* = 0.028] in the fully adjusted model. The fully adjusted model was considered well-adjusted in the light of the Hosmer-Lemeshow test (all *p* > 0.05) and is depicted in Figure 2.

**Table 3.** Short-term outcomes in participants according to categories of S′.

	All (732) *n* (%)	Group 1 (S′<10cm/s) (*n* = 181)	Group 2 (S′ 10–11 cm/s) (*n* = 290)	Group 3 (S′ >11 cm/s) (*n* = 261)	*p* Values
All-cause mortality	23 (3.1)	5 (2.8)	8 (2.7)	10 (3.8)	0.729
In-hospital	17 (2.3)	3 (1.7)	5 (1.7)	9 (3.4)	0.382
Post-discharge at 30 days	6 (0.8)	2 (1.1)	3 (1.0)	1 (0.4)	0.665
All-cause rehospitalization at 30 days	74 (10.1)	29 (16.0)	35 (12.1)	10 (3.8)* §	<0.001
HF	62 (8.5)	28 (15.5)	29 (10.0)	5 (1.9)* §	<0.001
Non-HF	12 (1.6)	1 (0.5)	6 (2.1)	5 (1.9)	0.463

HF: heart failure.

*p < 0.017 vs. group 1.

^§^*p* < 0.017 vs. group 2.

**Table 4.** Odds ratios with the 95% confidence interval for distinct logistic regression analyses models for a composite outcome.

Categories of S′-wave velocity(cm/s) continuous variable				
Model	Group 1 (<10) *n* = 181	Group 2 (10–11) *n* = 290	Group 3 (>11) *n* = 261	*n* = 732
Crude model	3.66 (1.92–7.00)	2.40 (1.28–4.48)	1 (Reference)	0.83(0.75–0.92)
*p* Values	<0.001	0.006		<0.001
Model 1	4.30 (2.21–8.36)	2.47 (1.32–4.63)	1 (Reference)	0.81(0.73–0.90)
*p* Values	<0.001	0.005		<0.001
Model 2	2.69 (1.30–5.57)	2.10 (1.08–4.09)	1 (Reference)	0.88(0.79–0.99)
*p* Values	0.008	0.030		0.032
Model 3	2.90 (1.33–6.31)	2.18 (1.10–4.33)	1 (Reference)	0.87(0.77–0.99)
*p* Values	0.007	0.026		0.028

Model 1: Adjusted for age and sex.

Model 2: Further adjusted for first admission, NYHA class IV, hypertension, DCM, A fib/A flutter, serum sodium, serum albumin, SBP, and β-blockers.

Model 3: Further adjusted for LVEF, LV EDD, RA diameter and RV diameter.

**Figure 3.**
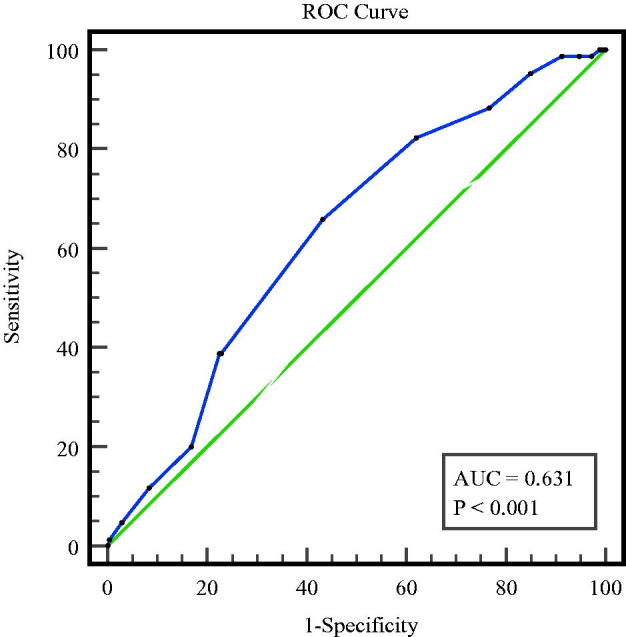
**ROC** curves of the ability of RV-S’ to predict a composite outcome.

To evaluate the predictive performance of RV-S' for a composite outcome, the area under curve (AUC) was calculated, which was 0.631 (95%CI: 0.573**–**0.690, *p* < 0.01) (Figure 3). In addition, AUCs for LVEF, PASP, E/e′ and LA size were 0.588 (95%CI: 0.524–0.651), 0.605 (95%CI: 0.539–0.671), 0.633 (95%CI: 0.562–0.703), and 0.667 (95%CI: 0.606–0.729), respectively. However, the differences of AUCs between S′ and the other parameters were not statistically significant. Detailed information is summarized in Table 5.

**Table 5.** Comparison of AUROCs between the S’ and other echocardiographic parameters for predicting a composite outcome.

Comparison	Difference between AUROCs	95%CI	Z-statistic	*P*-value
S’ versus LVEF	0.0435	−0.0312-0.118	1.141	0.2538
S’ versus PASP	0.0052	−0.08-0.0904	0.120	0.9043
S’ versus E/e’ septal	0.0162	−0.0663-0.0986	0.384	0.7009
S’ versus LA diameter	0.0359	−0.0376-0.109	0.957	0.3384

### Subgroup analysis

LVEF provides important information for HF management. We therefore examined the associations between RV-S′ and the risk of a composite outcome stratified by LVEF. In LVEF ≥ 40%, S′ was inversely associated with the risk of a composite outcome [<10 cm/s OR 4.52, 95%CI:1.31–15.60; 10–11 cm/s OR 2.78, 95% CI: 1.05–7.39; *p* for trend = 0.011] in comparison with S′ at >11 cm/s (Table 6).

**Table 6.** Odds ratios with the 95% confidence interval for distinct logistic regression analyses models for a composite outcome, by LVEF.

Categories of S’ (cm/s)	LVE*F* < 40%, *n* (306)	LVE*F* ≥ 40% , *n* (426)
	Adjusted^a^ OR (95%CI)	Adjusted^a^ OR (95%CI)
Group 1 (<10)	1.44 (0.54 − 3.85)	4.52 (1.31–15.6)
Group 2 (10–11)	1.39 (0.51–3.75)	2.78 (1.05–7.39)
Group 3 (>11)	1 (Reference)	1 (Reference)
*p* For trend	0.487	0.011

OR: odds ratio; LVEF: left ventricular ejection fraction.

^a^adjusted for age, sex, first admission, NYHA class IV, hypertension, DCM, A fib/A flutter, serum sodium, serum albumin, SBP, β-blockers, LV EDD, RA diameter and RV diameter.

### Sensitivity analysis

We submitted the LA size, not imputed or multiple imputed PH/PASP, and E/e′ data into model 3 for further adjustment as described above. As expected, the results were basically the same as before the adjustment. Detailed information is presented in Table 7.

**Table 7.** Sensitivity analyses: logistic regression models of a composite outcome among different groups of S′.

	A total number of patients (*n* = 732)					
		Adjusted^a^ OR	Adjusted^b^ OR	Adjusted^c^ OR	Adjusted^d^ OR	Adjusted^e^ OR
Categories of S′ (cm/s)	n	(95%CI)	(95%CI)	(95%CI)	(95%CI)	(95%CI)
Group 1 (<10)	181	2.90 (1.33–6.31)	3.38 (1.34–8.50)	2.62 (1.18–5.80)	3.38 (1.35–8.50)	2.60 (1.18–5.75)
Group 2 (10–11)	290	2.18 (1.10–4.33)	1.91 (0.86–4.24)	2.05 (1.02–4.10)	1.92 (0.87–4.25)	2.05 (1.02–4.10)
Group 3 (>11)	261	1 (Reference)	1 (Reference)	1 (Reference)	1 (Reference)	1 (Reference)
*p* For trend		0.006	0.01	0.015	0.01	0.015

OR: odds ratio.

^a^adjusted for age, sex, first admission, NYHA class IV, hypertension, DCM, A fib/A flutter, serum sodium, serum albumin, SBP, β-blockers, LVEF, LV EDD, RA diameter and RV diameter.

^b^further adjusted for LA diameter, not-imputed PH and E/e′.

^c^further adjusted for LA diameter, multiple imputed PH and E/e′.

^d^further adjusted for LA diameter, not-imputed PASP and E/e′.

^e^further adjusted for LA diameter, multiple imputed PASP and E/e′.

## Discussion

In this study, we identified a significant inverse association between PW-DTI-derived tricuspid lateral annular peak systolic velocity and the risk of poor short-term prognosis in patients with ADHF. As far as we know, this is the first comprehensive evaluation of tricuspid annular systolic velocity among patients with ADHF. More specifically, all patients with S′≤11 cm/s (groups 1 and 2) had a significant higher risk of short-term all-cause mortality or 30-day HF rehospitalization, with more adverse events detected across the escalating impaired S’ severity. This was independent of potential confounders, such as age, sex, comorbidities, physical examinations, lab tests, baseline medications, and echocardiographic parameters. These findings imply that S’ may serve as a useful tool to identify the patients with ADHF that are at a high risk of adverse events, and thus the evaluation of S′ should be reckoned as a part of the comprehensive assessment of this condition.

The structure of the right ventricle is characterized by a thin wall and irregular shape, with a high afterload sensitivity. Therefore, the Simpson’s method, which is commonly used to calculate the LVEF and is one of the significant indices for evaluating the LV systolic function, is not applicable to assess the right ventricular ejection fraction (RVEF) in clinical practice. Several previous studies have verified a correlation between RVEF and adverse outcomes in different HF populations using various methods of RVEF assessment, which are not widely used in clinical practice owing to the disadvantages we mentioned above [25–28]. Currently, S′ of tricuspid annulus has been shown to correlate well with other methods on assessing the RV contractile ability alongside its additional merits including ease in performance and noninvasiveness [20].

Till date, cumulative studies have confirmed that RV dysfunction is a prognostic factor of undesirable outcomes across a string of cardiac conditions. In 1998, De Groote et al. corroborated that RVEF is an independent predictor of survival in patients with moderate CHF. In 2010, Meyer et al. proved that baseline RVEF <20% is a significant independent predicator of mortality and HF hospitalization in systolic HF. In 2014, Melenovsky et al. reported that the right heart dysfunction is common in HF with preserved ejection fraction (HFpEF) and is driven by the RV contractile disability and afterload mismatch from PH. A recent study demonstrated that lower RVEF is associated with incident HF or death and was independent of LVEF among individuals free of HF [29]. However, data on the relevance of RV systolic dysfunction on the prognosis of ADHF remains limited. The frequency of RV contractile attenuation approached a quarter across all participants according to our present study. Moreover, it is associated with a poorer short-term prognosis of ADHF, with a heightened risk of short-term all-cause mortality or HF readmission reaching 190% (CI, 33–531%), when compared with patients with S′>11 cm/s. Furthermore, participants with S′ (10–11 cm/s) also presented more than twice the risk of a composite outcome in comparison with the group with the highest S′, while the LVEF has been the primary focus for patient with HF since it provides useful information for HF management. However, it is obvious that EF cannot identify a homogeneous group of patients [30]. Additionally, previous studies have shown that both short and long-term prognosis of different types of patients with HF (HFrEF, HFmrEF, or HFpEF) varies considerably [31–34]. Our study showed that LVEF was not independently associated with the outcome in multivariable analyses. On the contrary, both low and intermediate S′ categories presented an increased risk of a composite outcome after the adjustment of LVEF, which indicates that a low S′ may be a significant predictor of adverse events among patients with ADHF.

Few investigators reported that PH correlates with the S′ of tricuspid annulus below 12 cm/s. However, this finding has not been verified using right cardiac catheterization. As a complex and often misunderstood disorder, PH mostly results from the left cardiac conditions such as HF with reduced ejection fraction (HFrEF) and HFpEF [35,36]. Our findings demonstrated that patients in group 1 (S′<10 cm/s) truly exhibited a significant higher rate of PH (PASP > 40 mmHg). However, there was no statistical difference between the other two groups of participants in terms of the incidence of PH. Additionally, we found that few participants with specific conditions, such as congenital heart disease and valvular heart disease, possessed a normal range of S′, inspite of having severe PH. Nonetheless, the inverse relationship between S′ and risk of recent adverse events remains unchanged regardless of the adjustment of PH. Future investigators should pay more attention to the ambulatory monitoring of pulmonary pressure gauged by both non-invasive methods, such as echocardiogram, or invasive procedures, such as right heart catheterization. These considerations can possibly provide prognostic value for patients with HF.

Our findings also spotted that the systolic dysfunction of right and left ventricle coexist in a large portion of patients with DCM due to its highest prevalence in group 1. Likewise, ORs and 95% CI (S’) are consistent with the results before and after adjusting the DCM.

Our present study with its relatively large study population (*n* = 732) afflicted by all types of ADHF (LVEF ranging from 12 to 75%) expands previous awareness by displaying an inverse correlation between S’ and risk of short-term all-cause mortality or 30-day HF readmission. There might be other explanations for the relationship; however, the findings are intriguing enough to warrant further investigations that focus largely on the comprehensive echocardiographic parameters of RV systolic function both in and out of the hospital setting. In this way, we would be capable of discerning high-risk patients with ADHF as well as optimizing preventive interventions to improve the prognosis of people with ADHF.

## Limitations

In an observational study, it is impossible to eliminate residual confounding factors that could bias our results. However, in our study, potential confounders were attentively chosen, considering the number of events available, and none of these changed our findings following multivariable analyses. Due to the current medical status across China, we have gathered limited echocardiographic parameters for evaluating the systolic dysfunction of right ventricle, such as the absence of fractional area change (FAC) and tricuspid annular plane systolic excursion (TAPSE). However, we applied few major echocardiographic variables in the multivariable and sensitive analyses, and the results are consistent from start to finish. Lastly, we chose the Chinese participants as the study population, and this may limit the generalizability of our findings to other ethnicities.

## Conclusions

Inspite of potential confounders, a more impaired tricuspid lateral annular peak systolic velocity is associated with a poorer short-term prognosis of patients with ADHF. Tricuspid annular systolic velocity could be an independent predictor of poor short-term prognosis in ADHF, and thus it should be considered in patients with ADHF at admission.

## Data Availability

The data used to support the findings of this study are available from the corresponding author upon request.
